# Molecular and functional characterization of a new 3′ end KIT juxtamembrane deletion in a duodenal GIST treated with neoadjuvant Imatinib

**DOI:** 10.18632/oncotarget.19341

**Published:** 2017-07-18

**Authors:** Vittorio Perfetti, Erik Laurini, Suzana Aulić, Maurizio Fermeglia, Roberta Riboni, Marco Lucioni, Elena Dallera, Sara Delfanti, Luigi Pugliese, Francesco Saverio Latteri, Andrea Pietrabissa, Sabrina Pricl

**Affiliations:** ^1^ Internal Medicine, Ospedale SS Annunziata-ASST Pavia and Department of Molecular Medicine University of Pavia, 27100 Pavia, Italy; ^2^ Molecular Simulation Engineering (MOSE) Laboratory, Pharmaceutical and Molecular Biology Division, DEA, University of Trieste, 34127 Trieste, Italy; ^3^ Department of Molecular Medicine and Anatomic Pathology Section, Fondazione IRCCS Policlinico San Matteo, 27100 Pavia, Italy; ^4^ Department of Oncology and Hematology, Fondazione IRCCS Policlinico San Matteo, 27100 Pavia, Italy; ^5^ Department of Surgery, General Surgery II, University of Pavia and Fondazione IRCCS Policlinico San Matteo, 27100 Pavia, Italy; ^6^ General Surgery, Ospedale Cannizzaro, 95126 Catania, Italy

**Keywords:** KIT, GIST, novel deletion mutation, Imatinib, neoadjuvant treatment

## Abstract

Gastrointestinal stromal tumors (GISTs) are the most common mesenchymal tumors of the gastrointestinal tract. GISTs express the receptor tyrosine kinase KIT, and the majority of GISTs present KIT gain-of-function mutations that cluster in the 5′ end of the receptor juxtamembrane domain. On the other hand, little information is known about GISTs carrying mutations in the 3′ end of the KIT juxtamembrane domain. Here we report and discuss a clinical case of localized duodenal GIST whose molecular characterization revealed the presence of a new 21 nucleotide/7 amino acid deletion in the 3′ end of KIT juxtamembrane domain (Δ574–580). The patient was treated with Imatinib at standard regimen dose (400 mg/day), and responded well as the original tumor mass reduced, ultimately allowing conservative surgery. In line with these clinical evidences computer simulations, biophysical techniques and *in vitro* experiments demonstrated that the receptor tyrosine kinase KIT carrying the Δ574–580 mutation displays constitutive phosphorylation, which can be switched-off upon Imatinib treatment. In addition, results from this study showed that a clinical useful procedure, neoadjuvant treatment, can occasionally be of value for the understanding of the molecular pathogenesis of GIST.

## INTRODUCTION

Gastrointestinal stromal tumors (GISTs) are a relatively rare entity, accounting for less than 1% of GI tumors; nonetheless, they represent the most common mesenchymal tumors of the GI tract. GISTs arise from the muscle layer and are usually found in the stomach (60–70%), the proximal small intestine (25–30%), but can occur anywhere along the GI tract, exceptionally in the esophagus [1, 2]. In nearly 80–90% of GISTs, the oncogenic driver is a gain-of-function genetic alteration (mutations) in the receptor tyrosine kinase (RTK) KIT and it is now widely believed that GISTs arise from KIT-expressing interstitial cells of Cajal or their precursors [3]. The vast majority of *KIT* mutations (60–70%) are in-frame deletions (other include missense mutations (20–30%) and internal tandem duplications) clustered in the 5′ end of KIT juxtamembrane (KIT-JM) domain (exon 11) between Q550 and E561. Alterations in the 3′ end distal part of KIT-JM are rarely reported, and these include missense point mutations in codon L576, in-frame deletions, and rare internal tandem duplications of 1 up to more than 20 codons that are more often observed in gastric GISTs and associated with a favorable outcome [4]. Functional and molecular characterization of these rare 3-end variants is lacking. Other alternative mutational “hotspots” in KIT extracellular (exon 9, 18% of cases) and kinase (exon 13 and 17, less than 2%) domains have been identified in the GISTs that are negative for the exon 11 mutation [5, 6]. In the absence of KIT mutations, GISTs can harbor mutations of the *PDGFRA* gene (5–15% of cases), mainly located at exons 12 and 18, which are homologous with *KIT* exons 11 and 17 [7, 8]. The remaining cases (12–15%) lack *KIT* and *PDGFRA* mutations (*KIT* and *PDGFRA* wild-type GISTs), but these may include *BRAF* mutations (3%), loss of function of succinate dehydrogenase (SDH) complex (3%), and *NF-1* mutations [9].

Seventy five % of GISTs are less than 4 cm of size, and it is recommended that tumors greater than 2 cm of size should undergo surgical resection [10]. In particular, approximately 20–25% of gastric and 40 to 50% of small intestinal GISTs are clinically malignant, metastases commonly develop in the abdominal cavity and liver, and may develop 10 to 15 years after primary surgery necessitating long-term clinical follow-up [2].

Most GISTs are localized and are managed by surgery alone; however, locally advanced or metastatic cases, as well as high-risk operated GISTs, require systemic therapy with Imatinib, a small molecule tyrosine kinase inhibitor. Response to Imatinib correlates with the presence and type of RTK KIT mutations. GISTs with the most common 5′ end KIT exon 11 mutations show the highest response rates, whereas the responsiveness of exon 9 KIT mutations appears to be sensitive to increased drug concentrations [11]. On the other hand, information on Imatinib activity in case of the rare 3′ end KIT-JM mutations is lacking. Neoadjuvant Imatinib is now considered a valuable option for treating KIT-mutated GISTs [12]; it can render a locally advanced GIST resectable, allow to perform less invasive procedures or to promote preservation of function, especially if the tumor is located in an anatomically difficult position, as in case of the rare subset of GISTs that arise in the duodenum (2–5% of cases) [13]. Furthermore, neoadjuvant treatment has the potential to be of value for tumor biology, providing a way to gain *in vivo* information on Imatinib sensitivity in case of new mutations of undetermined functionality.

Under this perspective, in this work we report and discuss a clinical case of localized duodenal GIST whose molecular characterization revealed the presence of a new 21 nucleotide/7 amino acid deletion in the 3′ end of KIT-JM domain (Δ574–580). The patient was treated with Imatinib at standard regimen dose (400 mg/day), and responded well as the original tumor mass reduced, ultimately allowing conservative surgery. In line with these clinical evidences computer simulations, biophysical techniques and *in vitro* experiments demonstrated that the TKR KIT carrying the Δ574–580 mutation displays constitutive phosphorylation, which can be switched-off upon Imatinib treatment.

## RESULTS

### Patient clinical history

**Figure 1:**
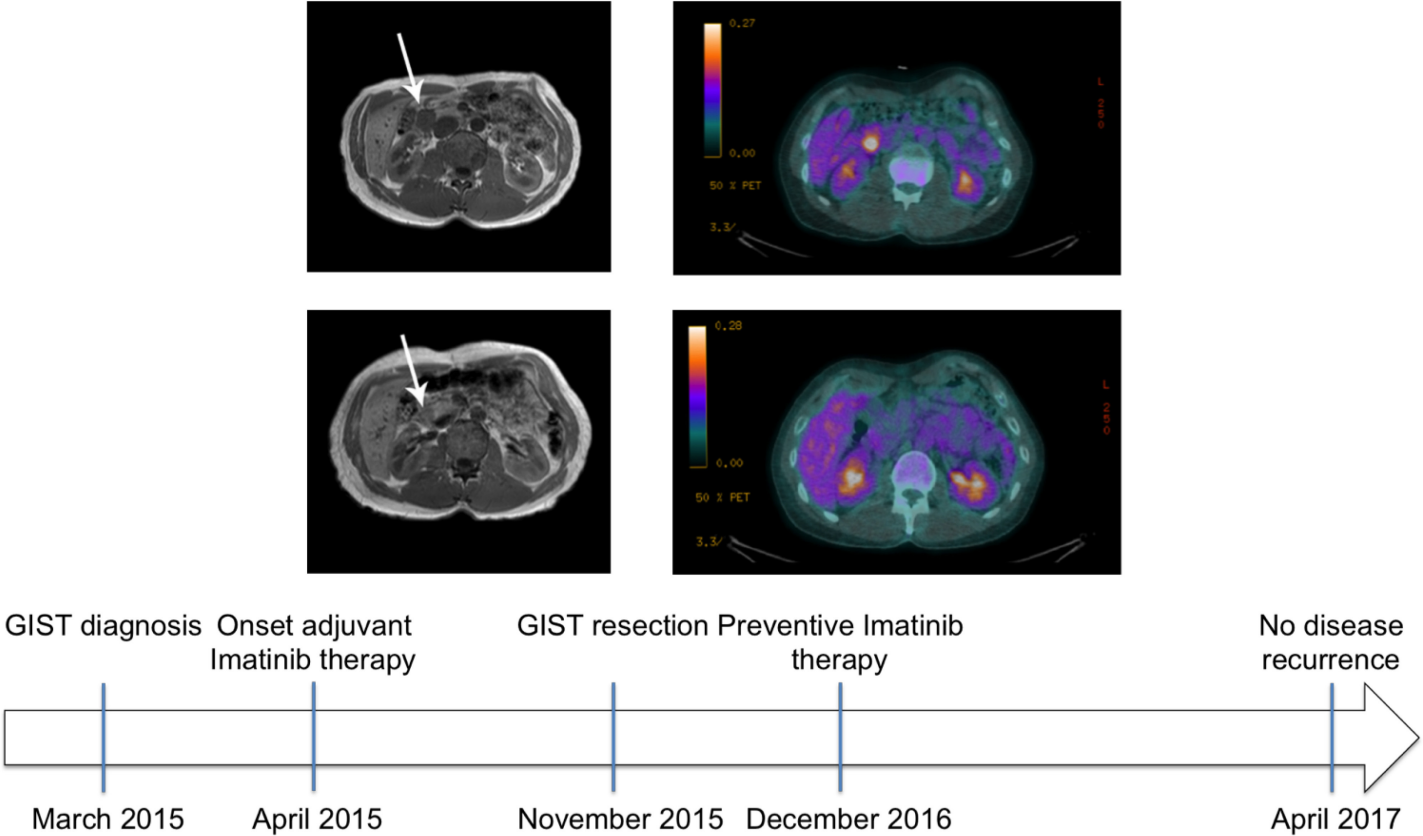
MRI and CT scan of patient at time of diagnosis and after Imatinib treatment Top panel: Contrast-enhanced nuclear magnetic resonance of the abdomen (left) and whole body ^18^F-FDG PET/CT of the patient with a duodenal GIST (arrow) at time of diagnosis (right). The mass displayed contact with various structures (left) and manifested intense metabolic activity (right, SUV 6.3). Middle panel: Contrast-enhanced nuclear magnetic resonance (left) of the patient abdomen at time of post-neoadjuvant surgery. The mass was reduced in size allowing to perform segmental duodenectomy safely. Whole body ^18^F-FDG PET/CT of the patient (right) that after a single month neoadjuvant Imatinib showed absent metabolic activity, attesting optimal drug sensitivity. Bottom panel: patient history timeline.

On March 2015 a 33-year-old man with silent medical history presented at the emergency unit with acute GI tract hemorrhagic anemia. No other clinical signs or symptoms were present. A CT scan showed a lesion in the II duodenal tract and the patient was admitted. Endoscopy showed persistent bleeding from the submucosal ulcerated duodenal mass. Contrast-enhanced abdomen NMR detected an oval mass (3.4 × 2.2 × 3.1 cm) with regular margins, in the medial aspect of the duodenal wall, immediately distal to the papilla and extending to nearby pancreatic head, displaying contact with the inferior cava vein (Figure 1, top panel, left).

Cytology obtained via echoendoscopy was highly suggestive for GIST, spindle cell type. Molecular typing was not readily possible, clinical conditions prompted intervention and the patient was eager to start treatment. The mass was technically resectable but required pancreatic-duodenectomy, with consequent significant morbidity. Bleeding indicated significant growth. ^18^F-FDG-PET/CT scan showed intense metabolic activity (SUV 6.3) (Figure 1, top panel, right), as expected for most GISTs, providing a way for testing Imatinib activity after a short drug trial in the context of a neoadjuvant approach (despite limited information in duodenal GISTs, Imatinib-sensitive mutations involving exon 11 are not uncommon) [14]. Indeed, a multidisciplinary evaluation proposed neoadjuvant treatment at the standard 400 mg/day Imatinib dose, which was started on early April 2015. Concomitantly, the patient underwent also a complete cardiac evaluation. Routine liver and renal function tests were within normal range.

### Tumor histology

The duodenal lesion after Imatinib neoadjuvant treatment showed size reduction (2.5 × 1.5 × 1.5 cm); the lesion was of solid, white-yellowish appearance, entirely constituted by proliferation of spindle cells, with mild cellular atypia, and a storiform growth pattern (Supplementary Figure 1A). Immunophenotypic profile was: CD117/c-KIT +/− and DOG1 + (Supplementary Figure 1B and 1C), CD34 −/+, smooth muscle actin -, desmin -, H-caldesmon −/+, S100 -, CAM5.2 -. Focal necrosis (less than 5%) and plurifocal hemorrhagic areas were observed. Signs of tumor regression included fibrosis and hydropic degeneration and blood vessel hyalinization. Mitotic index was 1/50 HPF, Ki67 < 3%. The lesion was localized within the muscular layer with focal extension into the submucosa, and was partially surrounded by a fibrotic rim. Resection margins were clear of tumor tissue. Risk assessment was not feasible because neoadjuvant treatment alters the two critical parameters, size and mitotic index.

### KIT and PDGFRA molecular analysis

DNA sequencing of the duodenal GIST identified a new 21 codon in frame deletion c1718:1739del21 (p.T574_H580delTQLPYDH) encompassing the internal part of the juxtamembrane zipper region of KIT (JM-Z, 10 amino acids, residues 572–581), an area adjacent to the first trans-phosphorylation sites (Tyr 568 and Tyr 570) (see Supplementary Figure 2). As result of the deletion, the JM-Z consisted of just the 3 amino acids DPK. Interestingly, mutations described in this area include point mutations and internal tandem duplications [4]. No mutations were found in the *KIT* exons 9, 13 and 17, as well as in *PDGFRA* exons 12, 14 and 18 hot spots (Supplementary Figure 3).

### Patient evaluation after Imatinib treatment

After prolonged discussion with the patient and informed consent obtained, Imatinib was started at the usual daily dose of 400 mg. Patient was monitored for hemoglobin concentration and occult fecal blood. Counts were stable and at day +30 of treatment, metabolic activity was not detected at a second FDG-PET-CT (Figure 1, middle panel, right), attesting optimal Imatinib sensitivity. The treatment was protracted till conservative surgery could be planned (approximately 8 months later). At that time, CE-NMR documented a mass of 1.3 cm size (Figure 1, middle panel, left) that allowed conservative surgery. Segmental duodenectomy with end-to-end duodenal reconstruction [14] was performed with optimal outcome, tumor resection was radical. Imatinib was started again as soon as clinical conditions allowed, and this because tumor histology after neoadjuvant treatment cannot reliable predict risk category. The patient was free of recurrence at last contrast-enhanced CT scan of the abdomen (approximately 1.5 years after surgery, Figure 1, bottom panel).

### Computational results

**Figure 2:**
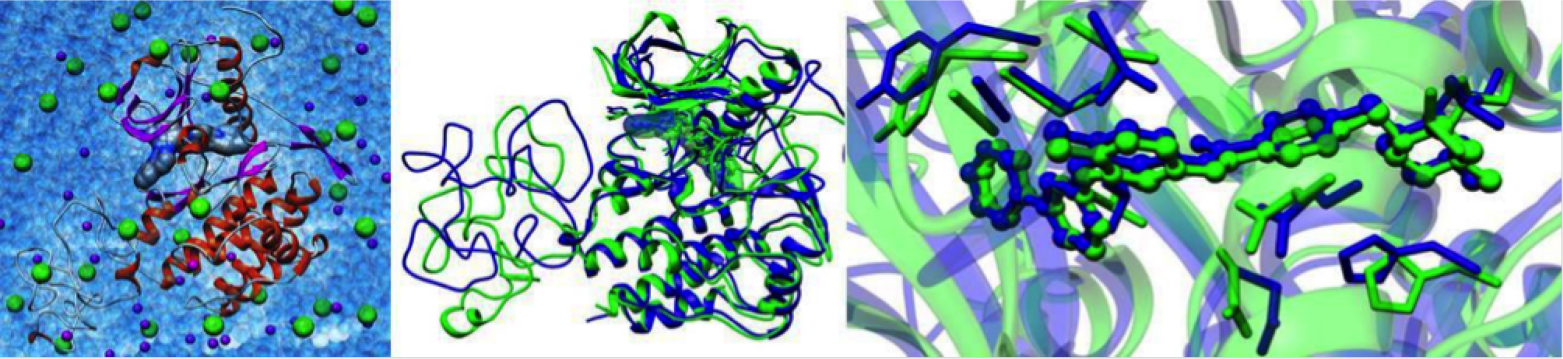
*In silico* analysis of Δ574–580 new KIT mutation (Left) The solvated 3D model of the Imatinib/Δ574–580 complex. The secondary structure of the protein is in ribbon representation (red, α-helices; magenta, β-sheets; gray, turns and coil, while Imatinib is portrayed via its van der Waals surface. Na^+^ and Cl^−^ ions and counterions are shown as purple and green spheres, respectively. Water molecules are depicted as transparent, light blue spheres. Hydrogen atoms are omitted for clarity. Superposition of MD equilibrated snapshots of Δ559 (green) and the Δ574–580 (blue) KIT in complex with Imatinib (center) and details of the corresponding Imatinib binding site (right).

The calculated free energy of binding values listed in upper part of Table 1 show that the newly reported Δ574–580 mutant receptor is endowed with an affinity toward Imatinib (ΔG_bind_ = −8.58 kcal/mol) slightly lower than that of the activating but Imatinib-responsive Δ559 isoform (ΔG_bind_ = −9.15 kcal/mol , ΔΔG_bind_ = −0.57 kcal/mol). Contextually, this deletion mutant seems to be provided with better affinity to the inhibitor compare to the WT receptor (ΔG_bind_ value is −8.19 kcal/mol, [15]). From the structural point of view, no significant perturbation of the overall 3D structure of the Imatinib/Δ574–580 complex is detected compared to the Imatinib responsive KIT Δ559 isoform (Figure 2). On the other hand, the T670I is found very resistant to the TK inhibitor, as expected (ΔG_bind_ = −6.38 kcal/mol).

**Table 1:** Binding free energies (ΔG_bind_) and binding free energy differences (ΔΔG_bind_) for the Δ559, Δ574–580, and T670I KIT receptors in complex with Imatinib and ATP

Imatinib binding	Δ574–580	Δ559	T670I
ΔG_bind_ (kcal/mol)	−8.58 ± 0.08	−9.15 ± 0.12	−6.38 ± 0.11
ΔΔG_bind_ (kcal/mol)	−0.57	-	−2.77
**ATP binding**	**Δ574–580**	**Δ559**	**T670I**
ΔG_bind_ (kcal/mol)	−24.02 ± 0.48	−24.20 ± 0.82	−25.14 ± 0.94
ΔΔG_bind_ (kcal/mol)	−0.18	-	+0.94

According to its definition (ΔΔG_bind_ = ΔG_bind,Δ559_ - ΔG_bind,mut_), negative values calculated for ΔΔG_bind_ indicate that the considered amino acid substitution at a given position of KIT is unfavorable in terms of imatinb/ATP affinity, whereas positive ΔΔG_bind_ values indicate that the considered mutation is favorable.

**Figure 3:**
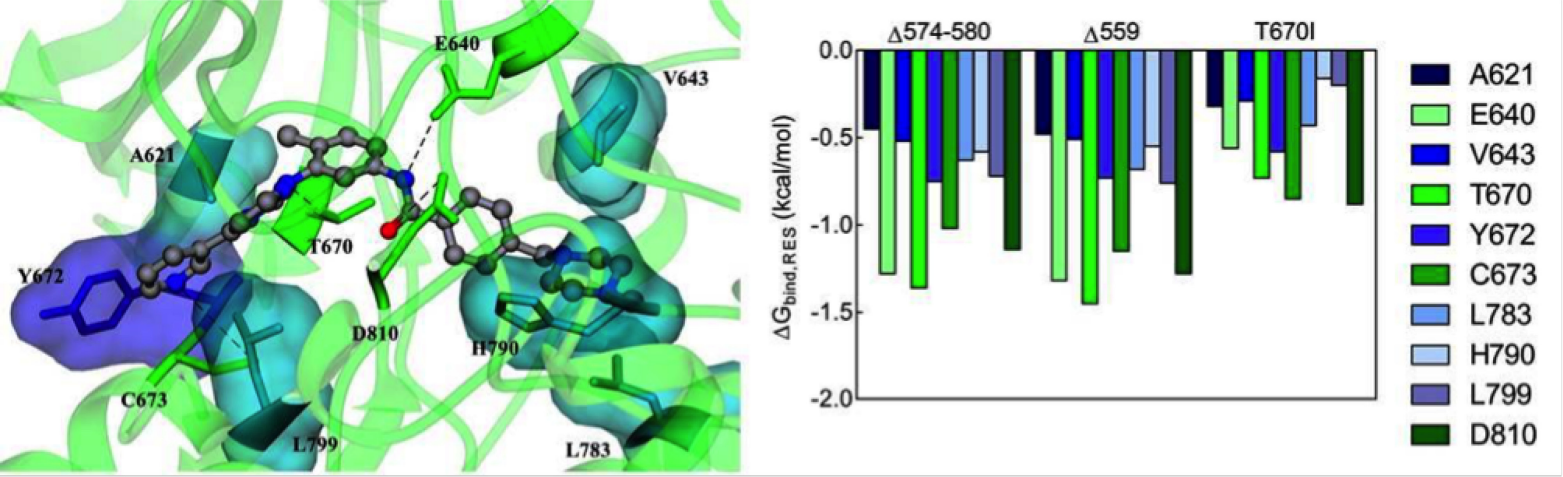
Molecular interactions between Imatinib and KIT mutants (Left) Equilibrated MD snapshot of the Δ574–580 KIT mutant receptor in complex with Imatinib. The image is a zoomed view of the receptor binding site. The ligand is portrayed as sticks-and-balls and colored by element (C, gray; N, blue; O, red), hydrogen atoms being omitted for clarity. The protein residues mainly involved in the interaction with the inhibitor are highlighted as colored sticks and labeled. H-bonds interactions are shown as dotted black lines. (Right) Comparison of per-residue binding energy decomposition for Imatinib in complex with Δ574–580, Δ559, and T670I KIT mutant receptors.

In order to gain further insight into the binding mode of Imatinib to Δ574–580 KIT mutant receptor, the energetic contribution (ΔG_bind,RES_) for those residues which afford a substantial contribution to the binding was calculated. As shown in the left panel of Figure 3, the major contribution to Δ574–580 KIT/Imatinib binding stems from the combined action of four hydrogen bonds involving the side chains of residues E640, T670, C673, and D810 and further stabilizing interactions provided by two clustered hydrophobic regions (HRI and HRII) of the receptor including residues: A621, Y672, and L799 (HRI) and V643, L783, and H790 (HRII), respectively. The strength of the hydrogen bonds network is ensued by the persistence and optimal average dynamics length (ADL) of these interactions, monitored during all the equilibrated MD simulation. Indeed, the ADL values for each residue involved fall in the typical range of the permanent hydrogen bond interactions (ADL_E640_ = 1.93 ± 0.01 Å; ADL_T670_ = 1.91 ± 0.03 Å; ADL_C673_ = 2.15 ± 0.04 Å; ADL_D810_ = 2.05 ± 0.01 Å). Such efficient intermolecular interaction scheme accounts for the most substantial favorable energetic term to the total drug binding free energy (ΔG_bind_ = −8.58 kcal/mol, Table 1), as these residues *per s*e afford a stabilizing contribution of -8.23 kcal/mol (Figure 3, right panel).

In the case of the Imatinib/KIT Δ559 complex, no meaningful differences are detected at the individual residue binding level (Figure 3, right panel), and the identified interactions are in agreement with those described in previous works for these prototypical Imatinib-responsive KIT mutant [15–20]. Conversely, in the case of the Imatinib/KIT T670I assembly, the overall reduction of drug affinity cannot be attributed either to a single residue or to a particular cluster of binding site residues. Instead, a general decrease of the binding energy for all residues involved is predicted. This analysis confirms that the conformation adopted by the newly reported Δ574–580 KIT mutant and, in particular, of its inhibitor binding site, is comparable to the one characterizing the Imatinib-sensitive Δ559 KIT receptor; concomitantly, the quantitative examination of the essential molecular determinants for Imatinib binding provides a sensible explanation of the efficiency of this inhibitor against this new KIT juxtamembrane mutant.

The lower half of Table 1 lists the ΔG_bind_ value obtained *in silico* for the Δ574–580 KIT mutant. The calculated ΔG_bind_ values for the reference KIT variants Δ559 and T670I are also reported for comparison. As seen from these numbers, and from the related ΔΔG_bind_ values in the last row of the top half of Table 1, the Δ574–580 KIT isoform shows an affinity for ATP (ΔG_bind_ = −24.02 kcal/mol) utterly comparable to that of the Δ559 (ΔG_bind_ = −24.20 kcal/mol, ΔΔG_bind_ = −0.18 kcal/mol) and the T670I (ΔG_bind_ = −25.14 kcal/mol, ΔΔG_bind_ = +0.94 kcal/mol) mutants, supporting the activating character of these mutations [15–17, 19].

### Isothermal titration calorimetry (ITC)

**Figure 4:**
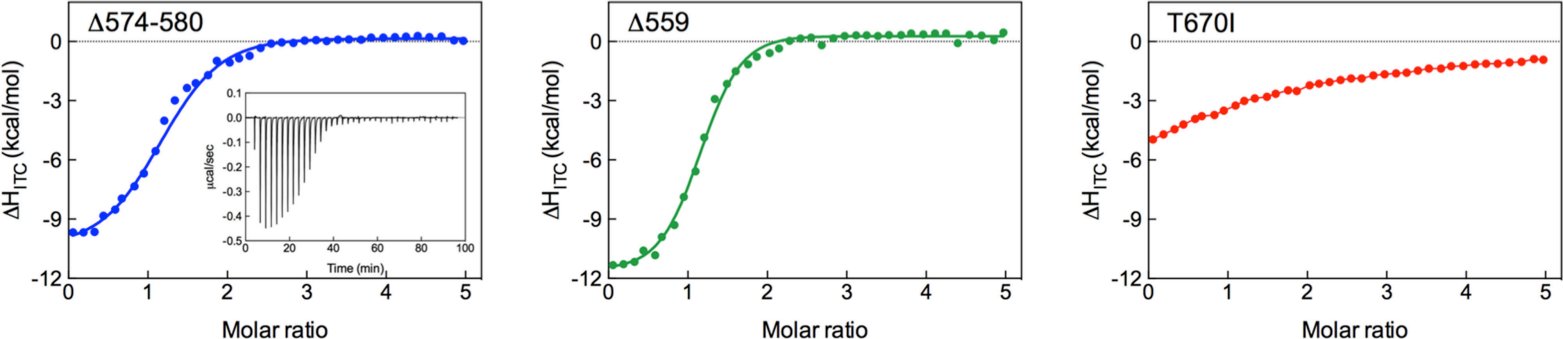
Isothermal titration calorimetry of KIT mutants with Imatinib ITC integrated heat curves for Imatinib binding to Δ574–580 (left), Δ559 (center), and T670I (right) KIT mutants. The inset shows ITC raw data for the tiration of Δ574–580 KIT mutant with Imatinib as an example. Solid curves represent data fitting with a 1:1 binding model.

Isothermal titration calorimetry (ITC) experiments were to validate *in silico* prediction of KIT mutants affinity for Imatinib via determination of the drug binding thermodynamics (i.e., ΔG_bind,ITC_ and its major enthalpic (ΔH_bind,ITC_) and entropic (-TΔS_bind,ITC_) components, the dissociation constant K_d,ITC_, and the binding stoichiometry *n*). Figure 4 shows the titration curves for Imatinib binding to Δ574–580, Δ559 and T670I KIT isoforms, while the relevant numerical results obtained from data fitting with a binding model that assumes one set of identical binding sites are displayed in Table 2.

**Table 2:** Thermodynamic parameters extracted from the calorimetric evaluation of unphosphorylated Δ574–580, Δ559, and T670I mutant KITs titrated Imatinib

KIT mutant	ΔGbind,ITC (kcal/mol)	ΔHbind,ITC (kcal/mol)	-TΔSbind,ITC (kcal/mol)	Kd,ITC (nM)	*n*
Δ574−580	−8.80	−10.73	1.93	359	1.12
Δ559	−9.41	−11.67	2.26	127	1.09
T670I	−6.18	−7.25	1.07	29450	0.96

Experiments were performed in triplicate at 25°C. Standard deviation values are within 1–3% for ΔH_bind,ITC_, 3–5% for K_d,ITC_, and 1–2% for *n*

As seen in Table 2, the stoichiometry of Imatinib binding to all KIT variants is approximately 1, confirming binding of one inhibitor molecule per RTK. The derived K_d,ITC_ confirm strong Imatinib binding to both deletion mutants (359 nM and 127 nM for Δ574–580 and Δ559 KIT, respectively). The ineffectiveness of Imatinib is obvious for T670I, with a K_d,ITC_ of approximately 30 μM. This analysis clearly demonstrate that Imatinib binds to the inactivated forms of the newly reported deletion mutant Δ574–580 with affinity comparable to that of the activating yet responding Δ559 isoform, and the loss of binding affinity of the inhibitor for the notoriously resistant T670I KIT variant. Finally, a comparison of the experimentally derived ΔG_bind,ITC_ values (Table 2) with the computer-predicted ones (ΔG_bind_, Table 1) reveals that the two data sets are in excellent agreement, ultimately yielding a direct validation of the *in silico* results presented above.

### Biological effects of the Δ574–580 mutation on KIT receptor activity

**Figure 5:**
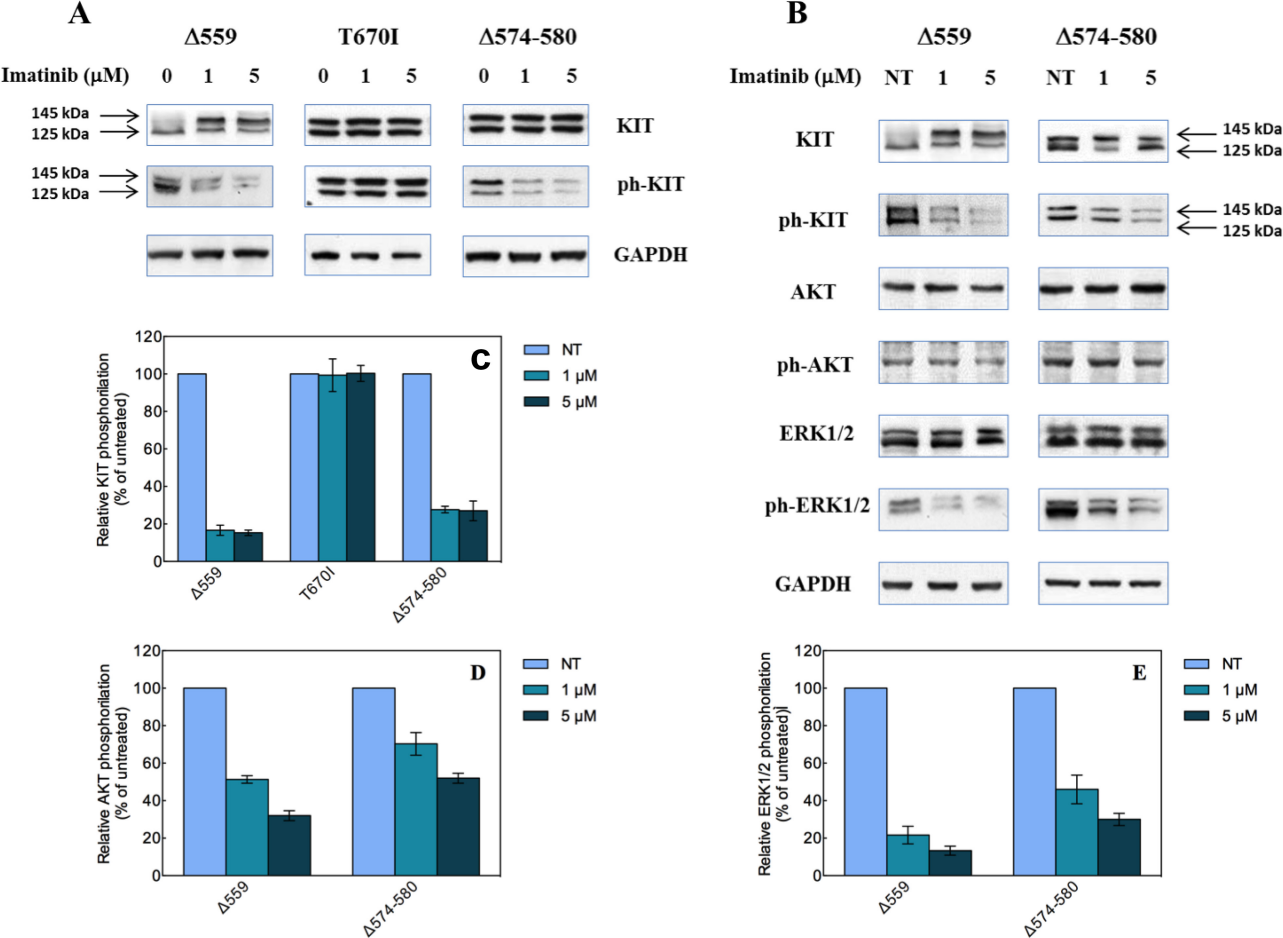
Biological effects of the Δ574–580 mutation on KIT receptor activity (**A**) Western blot analysis of HEK293 cells transfected with the indicated KIT mutant constructs, untreated (0 μM) or treated with 1 or 5 μM Imatinib. (**B**) Quantitative analysis of relative KIT phosphorylation (bottom) expressed as % ratio between phosphorylated and total KIT levels and referred to untreated cells. Data represent the average of three independent experiments ± the standard deviation. (**C**) Effect of Imatinib on AKT and ERK1/2 phosphorylation induced by expression of Δ559 and Δ574–580 KIT deleted mutants in transiently transfected HEK293 cells. Arrows indicate the 145 and 125 kDa KIT receptor forms. (**D** and **E**) Quantitative analysis of relative AKT (D) and ERK1/2 phosphorylation (E) expressed as % ratio between phosphorylated and total protein levels and referred to untreated cells. Data represent the average of three independent experiments ± the standard deviation.

Next, the effect of Imatinib on the activation of the Δ574–580 KIT mutant was examined. Again, the same study was conducted in parallel on the reference Δ559 and T670I KIT variants for comparison. Accordingly, the HEK293T cells previously transfected with the mutated *KIT* expression constructs were cultured in absence and in presence of 1 and 5 μM Imatinib, respectively. As shown in Figure 5A, all proteins are expressed and phosphorylated, and both the mature (145 kDa) and partially glycosylated (125 kDa) forms of the receptor could be detected by immunoblotting of the lysates. The new Δ574–580 KIT mutant reveals constitutive phosphorylation of both 145 and 125 kDa forms by the Western blot with anti-phospho-KIT antibodies. This behavior matches that observed for the well-defined Δ559 and T670I KIT variants, thereby confirming the constitutive KIT receptor-activating role of the Δ574–580 deletion, in agreement with the computational predictions reported in Table 1.

Also, again in accordance with *in silico* calculations/ITC determinations the newly reported KIT deletion variant is sensitive to Imatinib treatment at both concentrations (Figure 5A). Moreover, it exhibits a profile similar to that of the prototypical Imatinib-sensitive Δ559 KIT, in that the relative KIT phosphorylation levels are strongly reduced compared to the untreated cells (about 75%, Figure 5B). On the other hand, the T670I KIT mutant does not show any reduction of the KIT phosphorylation level in the presence of Imatinib, in line with its intrinsic Imatinib-resistant profile.

Furthermore, the effects of the new reported deletion on the typical signaling pathway triggered by KIT were investigated by Western blotting in absence and in presence of Imatinib. As shown in Figure 5C, phosphorylation of ERK1/2 and AKT is detectable in untreated cell expressing Δ559 or Δ574–580, thereby confirming the constitutive activation character of the new deletion KIT mutant. Interestingly, at both Imatinib concentrations (1 μM and 5 μM), the inhibition promoted by the inhibitor switches off the Δ574–580 KIT pathway, as both AKT and ERK1/2 phosphorylation is reduced (Figures 5D and 5E). As a similar effect is seen for the well-known Imatinib-sensitive Δ559 KIT isoform, these evidences support the Imatinib-sensitive behavior of this new KIT juxtamembrane variant, as anticipated by computer-assisted predictions and confirmed by isothermal titration calorimetry experiments.

## DISCUSSION

Resistance to tyrosine kinase inhibitors is a major problem in cancer targeted therapy. Gastrointestinal stromal tumors are not exception to this, in that the majority (nearly 85%) of GISTs patients present primary activating mutations in the gene encoding the mast/stem cell growth factor receptor KIT, a type III RTK that plays an essential role in the regulation of cell survival and proliferation, hematopoiesis, stem cell maintenance, gametogenesis, mast cell development, migration and function, and in melanogenesis. Most of the reported KIT mutations in GISTs (missense, deletion and deletion/insertion variants) cluster in the receptor juxtamembrane domain (residues 550–586), encoded by the exon 11 of the related *KIT* gene. According to the COSMIC database, the most often reported KIT-JM mutations are point substitutions at positions 557 (e.g., W557R/G/S/C, V559D/A/G, and V560D/A/G), thereby affecting the 5′ end of the KIT-JM domain. At the same time, the efficacy of the tyrosine kinase inhibitor Imatinib in GISTs has been linked to the capacity of this small molecule to inhibit these aberrant receptor-activating mutant KIT proteins. Alterations at the other extreme of the KIT-JM (3′ end) are much less frequent, and these include missense point mutations in codon L576, in-frame deletions, and rare internal tandem duplications of 1 up to more than 20 codons. The latter KIT variants are more often observed in gastric GISTs, and are associated with a favorable outcome [4].

In this work we presented and discussed a newly discovered 3′ end KIT-JM 7-residues deletion mutant Δ574–5870 (i.e., delTQLPYDG), found in a 33-year-old male patient with a rare duodenal GIST that was difficult to resect at time of presentation. Neoadjuvant Imatinib at standard regimen (400 mg/day), reduced the tumor mass to an extent that conservative surgery could be practiced. Molecular analysis revealed that no other mutations were present either in the *KIT* exons 9, 13 and 17, or in the *PDGFRA* exons 12, 14 and 18.

In line with the *in vivo* observations, computer-based simulations predicted that the new Δ574–580 KIT variant was indeed Imatinib-responsive, with an inhibitor affinity comparable to that of the known activating and Imatinib-responding Δ559 KIT isoform. In fact, the calculated ΔG_bind_ for this mutant is −8.58 ± 0.08 kcal/mol, corresponding to K_d_ or IC_50_ values (obtained from the fundamental relationship ΔG_bind_ = RTln K_d_ = RT ln IC_50_) of 516 nM, while the corresponding values of these quantities for the Δ559 KIT mutants are −9.15 ± 0.12 kcal/mol and 198 nM, respectively (Table 1). At the same time, a validation test performed on the Imatinib refractory T670I KIT mutant yielded a ΔG_bind_ value of -6.38 ± 0.11 kcal/mol, corresponding to a K_d_ > 21 μM (Table 1).

Isothermal titration calorimetry (ITC) experiments (Figure 4) performed on purified, unphosphorylated KIT constructs fully supported the *in silico* predictions, leading to ΔG_bind,ITC_ and K_d,ITC_ values −8.80 kcal/mol and 359 nM for the Δ574–580 KIT and −9.41 kcal/mol and 127 nM for the Δ559 KIT, respectively (Table 2). The experimental values of ΔG_bind,ITC_ and K_d,ITC_ for the T670I KIT mutant (−6.18 kcal/mol and > 29 μM, Table 2) was also found in agreement with the corresponding computed values (Table 1), thereby ultimately validating the computational strategy and results adopted in the present study.

Cell-based assays next confirmed the constitutive activation of the KIT receptor bearing the Δ574–580 mutation; concomitantly, the capacity of Imatinib to inhibit this mutant KIT phosphorylation was also verified. Importantly, the effect of Δ574–580 KIT inhibition by Imatinib shared similar effects to those observed in cells transfected with the Δ559 KIT mutant, for which a strong phosphorylation of both AKT and ERK1/2 was detected (Figure 5).

In summary, in the present study we reported and characterized a new 7-residue deletion mutation Δ574–580 in the rarely involved 3′end of tyrosine kinase receptor KIT juxtamembrane region, found in a 33-year-old male patient diagnosed with localized duodenal GIST who successfully underwent neoadjuvant imatinib treatment at standard dose (400 mg/day). In line with the *in vivo* observation of imatinib optimal sensitivity as attested by early complete metabolic response at FDG-PET, the results achieved by a combination of experimental and computational techniques indicate that the new Δ574–580 KIT mutant does not influence the overall sensitivity of KIT mutant toward Imatinib. Molecular simulations reveal that the deletion of 7 amino acids in the juxtamembrane domain of the receptor does not perturb the overall protein structure as well the binding region in which Imatinib is encased (Figures 2 and 3). Furthermore, the predicted affinity values of this KIT variant for ATP (Table 1) are in line with those calculated for two renown activating KIT mutations, i.e., the Δ559 and T670I KIT receptor [15–17, 19]. The molecular rationale yielded by the computational procedure is validated through ITC-based receptor/drug binding experiments and immunoblotting analysis of the effect of this new deletion on the intrinsic KIT activity and its response to Imatinib. Indeed, phosphorylation of the Δ574–580 KIT mutant is inhibited by Imatinib in a way similar to that observed for the Imatinib-sensitive Δ559 KIT control, whereas the kinase activity and thus receptor phosphorylation of the T670I, the prototypical Imatinib resistant KIT variant, is not inhibited at drug concentrations between 1 and 5 μM. The positive response to the kinase inhibitor of the Δ574–580 KIT form is further confirmed by the analysis of its signaling pathway in presence and in absence of Imatinib, according to which both AKT and ERK1/2 phosphorylation is indeed impaired at both Imatinib concentrations considered (Figure 5).

In addition, results from this study showed that a clinical useful procedure, neoadjuvant treatment, can occasionally be of value for the understanding of the molecular pathogenesis of GIST.

## MATERIALS AND METHODS

### KIT and PDGFRA molecular analysis

Mutation analysis of *KIT* exons 9, 11, 13, and 17, as well as *PDGFRA* exons 12, 14, and 18, was performed using direct sequencing of PCR products as described previously [21, 22]. Tumor cells comprised 98% of the cells in the sample used for gene sequencing. Three independent PCR experiments were performed to confirm sequencing results.

### Computational Details

The 3D Δ574–580 (delTQLPYDH) KIT mutant model was obtained starting from our optimized WT KIT model in complex with Imatinib [15–20], following a consolidated methodology [23–27]. The validated structures of the Δ559 and T670I KIT mutants were also considered as reference for Imatinib responsive and resistant TK isoforms, respectively [15–20]. Following the same procedure, the complexes of all proteins with ATP were obtained from the corresponding optimized WT/KIT ensemble [15–17].

Each protein/ligand system was solvated, gradually heated to 25°C, equilibrated and subjected molecular dynamics (MD) data collection runs to perform drug/protein free energy of binding analysis [28–29].

All simulations were carried out using the *Pmemd* modules of Amber 16 [30], running on our Mose25 CPU/GPU calculation cluster (see the Supporting Information appendix for full computational details).

### Cloning an KIT mutants purification for experimental Imatinib binding studies

Δ559, T670I, and Δ574–580 mutant KIT constructs were produced according to the established methodology described by Gajiwala et al. [31]. All mutant plasmids were sequenced to verify the success of mutagenesis experiments. The purified c-Kit protein was not phosphorylated as judged by routine mass spectrometry (data not shown). Full details are provided in the Supporting information appendix.

### Isothermal titration calorimetry (ITC) experiments

Calorimetric titrations were carried out on a MicroCal PEAQ-ITC calorimeter (Malvern, UK) at 25°C. Before titrations, all KIT mutant proteins used were buffer-exchanged into an identical lot of HBS buffer (10 mM Hepes pH7.5, 150 mM NaCl). Gel filtration was used to control buffer heat dilution effects. Each protein sample was thoroughly degassed before ITC experiments, and these were run in triplicate (see Supporting information appendix for details).

### Construction and transfection of mutated KIT

Δ559, T670I and the new variant Δ574–580 mutant KIT receptors were obtained as following a methodology reported in our previous work [15–17, 19]. Briefly, an expression vector carrying wild-type (WT) human complementary DNA (cDNA) for *KIT* (kind gift of Professor Y. Yarden, Weizmann Institute, Rehovot, Israel) was used to generate all mutated forms of *KIT* via site-directed mutagenesis using the commercial QuickChange Site-Directed Mutagenesis kit (Promega, Madison, WI), following manufacturer’s instruction. We then constructed mutant Δ559, in which amino acid 559 is removed from the juxtamembrane region by deleting nucleotides 1696 – 1698 from the portion of the exon 11-derived WT cDNA. For the T670I mutant, the second base of the T670 triplet codon ACA (i.e., cytosine 2030), was mutated to a thymine. Finally, the Δ574–580 mutant was obtained by removing nucleotides 1741–1761 from the corresponding portion of the WT cDNA. All plasmid inserts were sequenced after mutagenesis to verify their identity.

### Cell cultures and transfections

HEK293T cells were maintained in Dulbecco’s Modified Eagle’s Medium (DMEM) supplemented by 10% fetal calf serum, in 5% CO_2_ humidified atmosphere, and transfected by Lipofectamine 2000 (Invitrogen), according to the manufacturer’s instructions. Cells were harvested 48 h after transfection following overnight serum starvation. Cell lysates were produced in RIPA modified buffer, and Western blot analysis was performed as described in our previous work [15–17, 19]. Anti-KIT (clone H300) antibodies were purchased from Santa Cruz Biotechnology (Santa Cruz, CA, USA); antiphospho KIT (Y719, ref. 3391), AKT and phospho AKT (Ser473, ref. 9271) antibodies were obtained from Cell Signal Technology (Beverly, MA, USA) while ERK1/2 and phospho ERK1/2 (T202/Y204, ref. SAB4301578) antibodies were acquired from Sigma-Aldrich (St. Louis, MO, USA).

## SUPPLEMENTARY MATERIALS FIGURES AND REFERENCES



## Abbreviations

CT: computed tomography
^18^F-FDG-PET/CT: 2-deoxy-2-[fluorine-18]fluoro- D-glucose integrated with computed tomography
GIST: gastrointestinal stromal tumors
JM: juxtamembrane
NMR: nuclear magnetic resonance
RTK: receptor tyrosine kinase
SUV: standardized uptake value
3D: three-dimensional.
