# Supplementary material for: Molecular and functional characterization of a new 3′ end KIT juxtamembrane deletion in a duodenal GIST treated with neoadjuvant Imatinib
Source: Oncotarget. 2017 Jul 18;8(34):56158–67. doi: 10.18632/oncotarget.19341 (PMC5593551; doi:10.18632/oncotarget.19341)
Supplement: Supplementary file 1 [file oncotarget-08-56158-s001.pdf]

Molecular and functional characterization of a new 3′ end KIT juxtamembrane deletion in a duodenal GIST treated with neoadjuvant Imatinib

SUPPLEMENTARY MATERIALS

Computational details

The optimized structure of KIT was taken from our previous work (Tamborini et al., 2006a, 2006b; McAuliffe et al., 2008; Negri et al., 2009; Woodman et al., 2009; Conca et al., 2013). Mutations Δ559, T670I and Δ574-580 were introduced into the wild-type structure of KIT/imatinib complex by swapping the mutant residue into the specific site, according to a dedicated and well-validated procedure (Tamborini et al., 2006a, 2006b; McAuliffe et al., 2008; Negri et al., 2009; Woodman et al., 2009; Conca et al., 2013). Each protein/ligand system was relaxed in a box of TIP3P water molecules (Jorgensen et al., 1983). To achieve electroneutrality, a suitable number of neutralizing ions were added; further, the solution ionic strength was adjusted to the physiological value of 0.15 M by adding the required amounts of Na^+ and Cl^- ions. Each hydrated complex system was then gradually heated to 25°C and then equilibrated for 5 ns via molecular dynamics (MD) simulations under isobaric/isothermal conditions (i.e., NPT ensemble). Finally, each MD equilibrated system was subjected to 50 ns of NPT MD data collection runs to estimate the corresponding drug/protein free energy of binding (Pierotti et al., 2010; Pierotti et al., 2011).

All simulations were carried out using the *Pmemd* modules of Amber 16 (Case et al., 2016).

All energetic analysis was carried out for only a single MD trajectory of each KIT/imatinib complex considered, with unbound protein and substrate snapshots taken from the snapshots of that trajectory.

The affinity of the mutant KIT isoforms for imatinib and ATP, expressed as the value of the corresponding free energy of binding ΔG_{bind}, was estimated using the Molecular Mechanics/Poisson-Boltzmann Surface Area (MM/PBSA) method (Kollman et al., 2000, Puerotti et al., 2011). Accordingly, the binding free energy between two biological entities (e.g., a drug and its protein target) in a solvent was obtained as the sum of the interaction energy between the receptor and the ligand (ΔE_{MM}), the solvation free energy ($\Delta G_{so}l$), and the conformational entropy contribution (−TΔS), averaged over a series of snapshots from the corresponding MD trajectories:

$$\Delta G_{bind} = \Delta E_M M + \Delta G_{solv} - T\Delta S \quad (S1)$$

The ΔE_{MM} term in Eq. (1) can be obtained directly from the molecular mechanics interaction energies as:

$$\Delta E_{MM} = \Delta E_{int} + \Delta E_{vdW} + \Delta E_{ele} \quad (S2)$$

where ΔE_{int}, ΔE_{vdW}, and $\Delta E_{el}e$ are the internal, van der Waals and electrostatic components of the nonbonded interaction energy, respectively. Since in this work we adopted the "single trajectory protocol", then $\Delta E_{int} = 0$ in Eq. (S2).

The second term Eq. (S1), the solvation energy ΔG_{sol}, can also be partitioned into two different contributions:

$$\Delta G_{solv} = \Delta G_{PB} + \Delta G_{NP} \quad (S3)$$

The polar term of ΔG_{solv}, ΔG_{PB}, was estimated by solving the Poisson-Boltzmann equations numerically and calculating the electrostatic energy according to the electrostatic potential. In these calculations, the interior and exterior dielectric constant values were set equal to 1 and 80, respectively. A grid spacing of 0.5 per Å, extending 20% beyond the dimensions of the solute, was employed. The value of nonpolar component of ΔG_{solv}, ΔG_{NP}, was calculated using the following relationship (Sitkoff et al., 1994):

$$\Delta GNP = \gamma \times SA + \beta \quad (4)$$

in which $\gamma = 0.00542$ kcal (mol Å2)$^{-1}$, $\beta = 0.92$ kcal/mol, and SA is the molecular surface area estimated by means of the MSMS software (Sanner et al., 1993).

The change in solute entropy upon association (−TΔS in Eq. (S1)) was evaluated using the Nmode module of Amber 16. The normal-node analysis was performed for the minimized structures of the complexes, KIT, and ligands using a distance-dependent dielectric constant $\varepsilon = 4r_{ij}$. In the first step of this calculation, an 8-Å sphere around the ligand was cut out from an MD snapshot for each ligand-protein complex. This value was shown to be large enough to yield converged mean changes in solute entropy. On the basis of the size-reduced snapshots of the complex, we generated structures of the uncomplexed reactants by removing the atoms of the protein and ligand, respectively. Each of those structures was minimized, using a distance-dependent dielectric constant $\varepsilon = 4r$, to account for solvent screening, and its entropy was calculated using classical statistical formulas and normal mode-analysis. To minimize the effects due to different

conformations adopted by individual snapshots we averaged the estimation of entropy over 50 snapshots.

A per-residue binding free energy decomposition (PRBFED) was performed exploiting the MD trajectory of each complex. This analysis was carried out using the MM/GBSA approach (Tsui and Case, 2000), and was based on the same snapshots used in the binding free energy calculation.

Cloning and KIT mutants purification for experimental Imatinib binding studies

Δ559, T670I, and Δ574-580 mutant KIT constructs were produced according to the established methodology described by Gajiwala et al. (2009). Accordingly, a construct encoding an N-terminal 6X-His tag + r3c protease site + KIT residues 544-976 was cloned into the baculovirus expression vector pVL1393 (Invitrogen). Proteins were expressed in Sf9 cells for 48 h; cells were then harvested and frozen (−80°C). Cellular lysates were obtained by treatment with 25 mM Tris (pH 7.4), 250 mM NaCl, 0.25 mM TCEP, and 20 mM imidazole. After binding to a Ni-NTA column, each protein was step-eluted using the same buffer employed for pellet lysing with 250 mM imidazole. Each peak eluted fraction was subjected to dialysis at 4°C for 12 h. Next, the His tag was cleaved with the r3C protease and removed by reloading the proteins on the Ni-NTA column. Each Flow-through was collected from the column and concentrated. Next, each protein was feeded to a Superdex-200 column equilibrated in 25 mM Tris (pH 7.4), 250 mM NaCl, 0.5 mM TCEP, and 1mM EDTA. The peak fractions were pooled, concentrated (up to 6 mg/ml) and quickly frozen.

Isothermal titration calorimetry (ITC) experiments

A solution of each purified KIT mutant protein (10 μM, sample cell) was titrated with 36 step-by-step injections (spacing 150 s) of 2 μL volume of Imatinib (250 μM, syringe). Solutions and buffer were degassed for 30 min at room temperature under stirring at 750 rpm prior to each experiment.

SUPPLEMENTARY REFERENCES

1. Case DA, Betz RM, Botello-Smith W, Cerutti DS, Cheatham III TE, Darden TA, Duke RE, Giese TJ, Gohlke H, Goetz AW, Homeyer N, Izadi S, Janowski P, et al. AMBER 2016, University of California, San Francisco (CA, USA). 2016.
2. Genheden S, Ryde U. The MM/PBSA and MM/GBSA methods to estimate ligand-binding affinities. Expert Opin Drug Discov. 2015; 10:449–461.
3. Kollman PA, Massova I, Reyes C, Kuhn B, Huo S, Chong L, Lee M, Lee T, Duan Y, Wang W, Donini O, Cieplak P, Srinivasan J, et al. Calculating structures and free energies of complex molecules: combining molecular mechanics and continuum models. Acc Chem Res. 2000; 33:889–897.
4. Jorgensen WL, Chandrasekhar J, Madura JD, Impey RW, Klein ML. Comparison of simple potential functions for simulating liquid water. J Chem Phys. 1983:79:926–935.
5. Pierotti MA, Negri T, Tamborini E, Perrone F, Pricl S, Pilotti S. Targeted therapies: the rare cancer paradigm. Mol Oncol. 2010; 4:19–37.
6. Pierotti MA, Tamborini E, Negri T, Pricl S, Pilotti S. Targeted therapy in GIST: *in silico* modeling for prediction of resistance. Nat Rev Clin Oncol. 2011; 8:161–170.
7. Sanner MF, Olson AJ, Spehner JC. Reduced surface: an efficient way to compute molecular surfaces. Biopolymers 1996; 38:305–320.
8. Sitkoff D, Sharp KA, Honig B. Accurate calculation of hydration free energies using macroscopic solvent models. J Phys Chem. 1994; 98:1978–1988.
9. Tamborini E, Negri T, Miselli F, Lagonigro MS, Pricl S, Pilotti S. Response of a KIT-positive extra-abdominal fibromatosis to imatinib mesylate and KIT genetic analysis. J Natl Cancer Inst. 2006a; 98:1583–1584.
10. Tamborini E, Pricl S, Negri T, Lagonigro MS, Miselli F, Greco A, Gronchi A, Casali PG, Ferrone M, Fermeglia M, Carbone A, Pierotti MA, Pilotti S. Functional analyses and molecular modeling of two c-Kit mutations responsible for imatinib secondary resistance in GIST patients. Oncogene 2006b; 25:6140–6146.
11. Tsui V, Case DA. Theory and applications of the generalized Born solvation model in macromolecular simulations. Biopolymers. 2000; 56:275–291.
12. Woodman SE, Trent JC, Stemke-Hale K, Lazar AJ, Pricl S, Pavan GM, Fermeglia M, Gopal YN, Yang D, Podoloff DA, Ivan D, Kim KB, Papadopoulos N, et al. Activity of dasatinib against L576P KIT mutant melanoma: molecular, cellular, and clinical correlates. Mol Cancer Ther. 2009; 8:2079–2085.

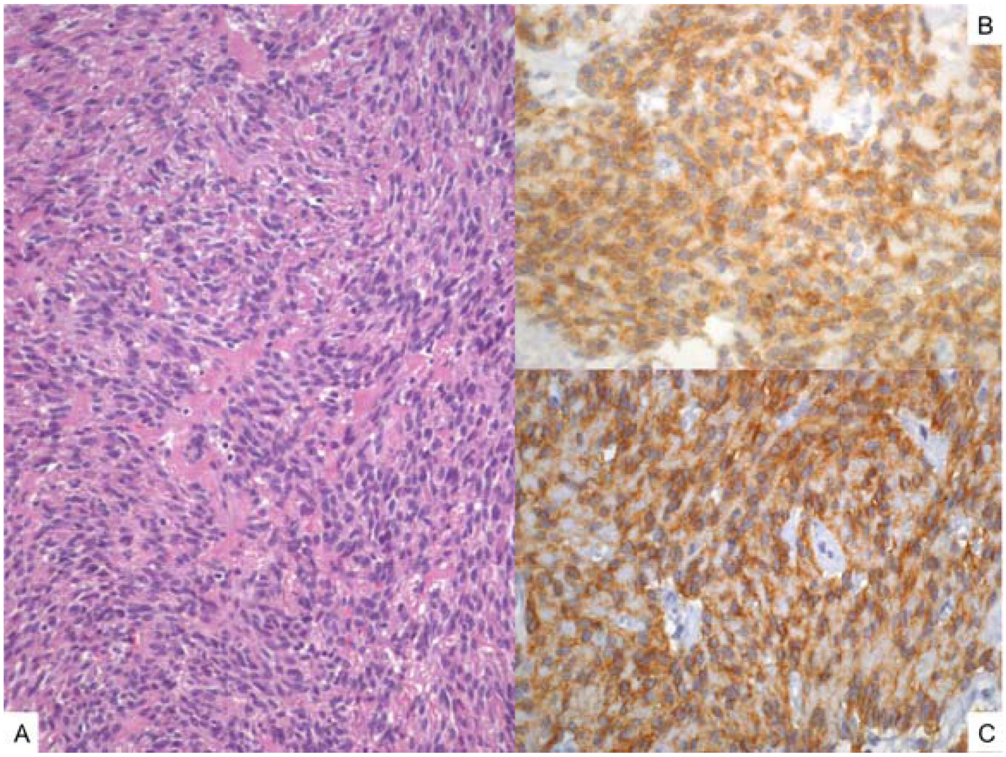

Supplementary Figure 1: Tumor histology: (**A**) Histopathologic features (HE, 200×): the tumor mainly consisted of spindle cells exhibiting a fascicular growth pattern. (**B**) Immunostaining for c-KIT/CD117 (400×): most tumor cells showed membranous and/or cytoplamatic positivity for CD117. (**C**) Immunostaining for DOG1 (400×): most tumor cells showed strong immunoreactivity for DOG1.

WT KIT EX 11 ACAGAAACCCATGTATGAAGTACAGTGGAAGGTTGTTGAGGAGATAAATGGAAACAATTA

PT KIT EX 11 ACAGAAACCCATGTATGAAGTACAGTGGAAGGTTGTTGAGGAGATAAATGGAAACAATTA

549 Q K P M Y E V Q W K V V E E I N G N N Y

WT KIT EX 11 TGTTTACATAGACCC**AACACAACTTCCTTATGATCA**CAAATGGGAGTTTCCCAGAAACAG

PT KIT EX 11 TGTTTACATAGACCCAACACAACTTCCTTATGATCACAAATGGGAGTTTCCCAGAAACAG

569 V Y I D P T Q L P Y D H K W E F P R N R

WT KIT EX 11 GCTGAGTTTTGGGAAAACCCTGGGTGCTGGAGCTTTCGGGAAGGTTGTTGAGGCAACTGC

PT KIT EX 11 GCTGAGTTTTGGGAAAACCCTGGGTGCTGGAGCTTTCGGGAAGGTTGTTGAGGCAACTGC

589 L S F G K T L G A G A F G K V V E A T A

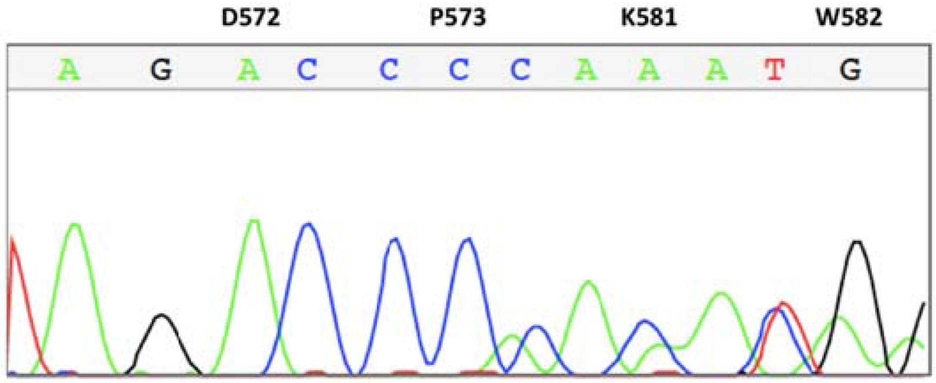

Supplementary Figure 2: Sequencing of exon 11 of WT and mutated *KIT*. Top panel: comparison between exon 11 of WT and patient *KIT*. Bottom panel: Patient *KIT* sequence (partial in length) comprising the region encoding for the newly reported mutation Δ574-580, sequence showing the in frame deletion mutation c1718:1739del21.

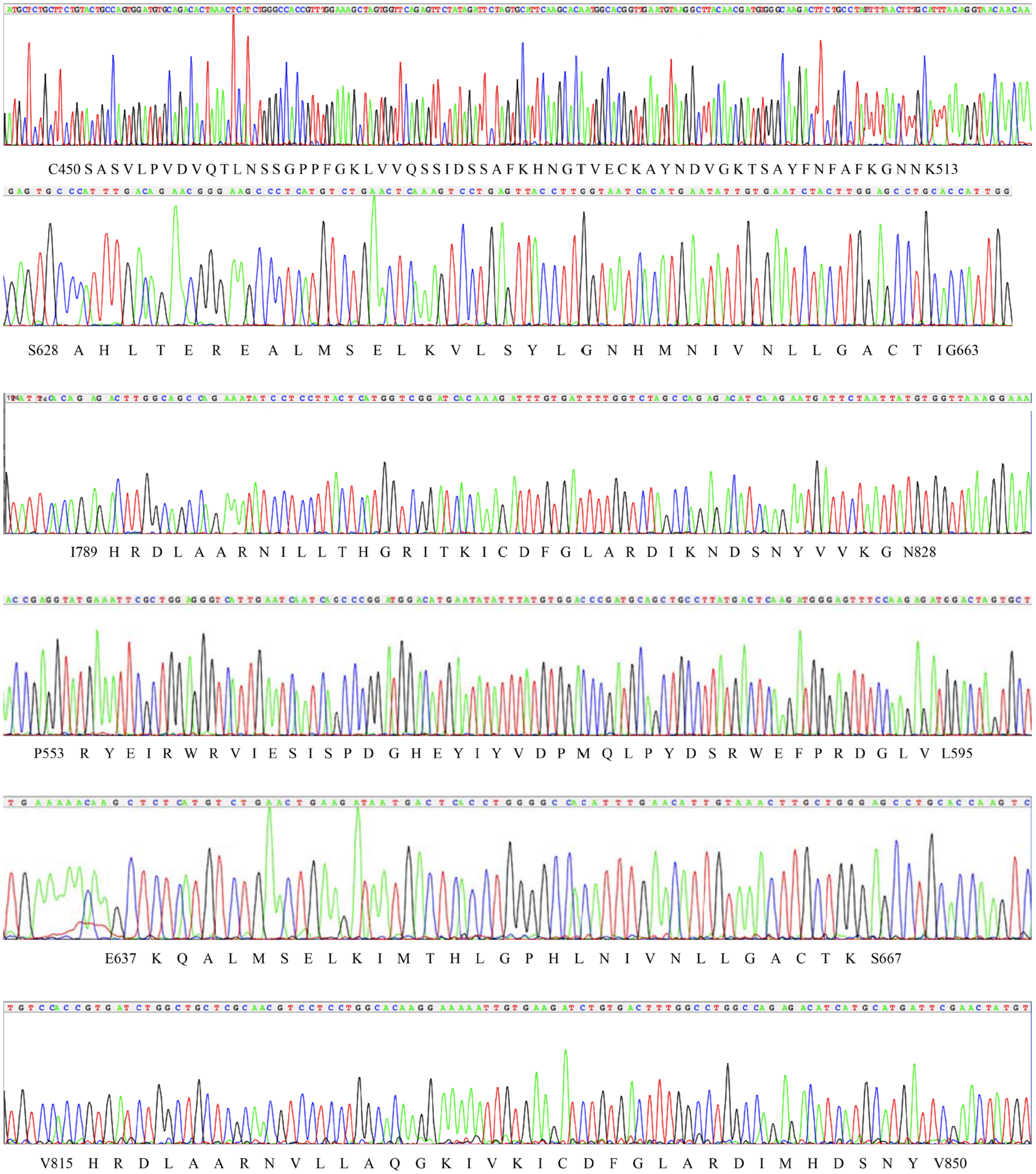

Supplementary Figure 3: Sanger sequencing of *KIT* exons 9, 13 and 17 hotspots (first three panels from top), and of *PDGFRA* exons 12, 14 and 18 hotspots (last three panels).
